# Through synapses to spatial memory maps via a topological model

**DOI:** 10.1038/s41598-018-36807-0

**Published:** 2019-01-24

**Authors:** Yuri Dabaghian

**Affiliations:** Department of Neurology, The University of Texas McGovern Medical School, 6431 Fannin St, Houston, TX 77030 USA

## Abstract

Various neurophysiological and cognitive functions are based on transferring information between spiking neurons via a complex system of synaptic connections. In particular, the capacity of presynaptic inputs to influence the postsynaptic outputs–the efficacy of the synapses–plays a principal role in all aspects of hippocampal neurophysiology. However, a direct link between the information processed at the level of individual synapses and the animal’s ability to form memories at the organismal level has not yet been fully understood. Here, we investigate the effect of synaptic transmission probabilities on the ability of the hippocampal place cell ensembles to produce a cognitive map of the environment. Using methods from algebraic topology, we find that weakening synaptic connections increase spatial learning times, produce topological defects in the large-scale representation of the ambient space and restrict the range of parameters for which place cell ensembles are capable of producing a map with correct topological structure. On the other hand, the results indicate a possibility of compensatory phenomena, namely that spatial learning deficiencies may be mitigated through enhancement of neuronal activity.

## Introduction

**Figure 1:**
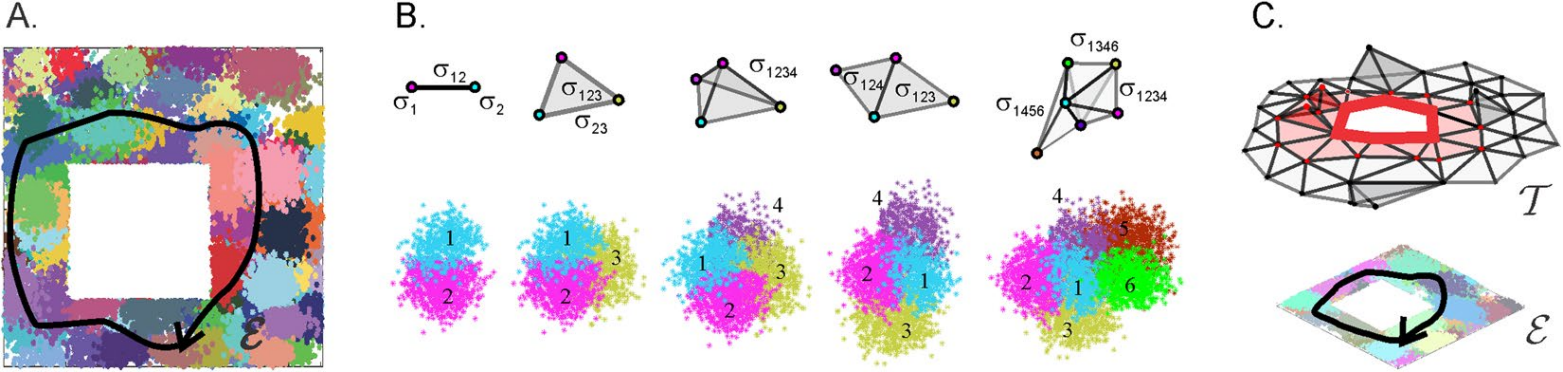
Place field map and nerve complex. (**A**) A place field map in a small 1*m* × 1*m* environment with one hole: spikes produced by different place cells are marked by dots of different colors. (**B**) In a schematic description of the place field map, each place field center gives rise to be a zero-dimensional vertex (0*D* simplex *σ*_*i*_); each pair of the overlapping place fields is represented by a link between corresponding vertices (1*D* simplex *σ*_*ij*_); a triple of overlapping place fields by a triangle (2*D* simplex *σ*_*ijk*_), four simultaneously overlapping place fields are represented by a solid tetrahedron, (3*D* simplex *σ*_*ijkl*_) etc. A less dense place field map is represented by two adjacent triangles—a simple example of a nerve complex. A place field map that consists of six place fields is represented by a nerve complex that consists of three tetrahedrons, *σ*_1234_, *σ*_1456_ and *σ*_1346_. (**C**) According to the Alexandrov-Čech’s theorem, the nerve complex construction for a place field map has the same topological shape as the underlying environment—in case of the map shown on panel A, the nerve complex ${\mathscr{N}}$ has one connected piece and contains a hole in the middle.

The location-specific spiking activity of the hippocampal neurons, known as place cells^1^, gives rise to an internalized representation of space–a cognitive map. Each place cell fires a series of action potentials in specific spatial region–its place field, so that the ensemble of such cells produces a “map” of the environment in which they are active (Fig. 1A). By construction, such a map defines the temporal order in which place cells fire as the animal explores the environment, and therefore it can be viewed as a geometric representation of the spatial memory framework encoded by the hippocampus^2,3^.

The exact nature of this framework is currently actively studied both computationally and experimentally. For example, it was demonstrated that if the shape of the environment gradually changes, then the place field map deforms in a way that preserves mutual overlaps, adjacencies, containments, etc., between the place fields^4–8^. This observation implies that the sequence in which the place cells fire during animal’s navigation remains invariant throughout the reshaping of the arena and suggests that the place cells do not represent precise geometric information, but a set of qualitative connections between portions of the environment–a topological map^8–11^.

From the computational perspective, the topological nature of the cognitive map suggests that the information transmitted via place cell spiking should be amenable to topological analyses. In our previous work^12–16^, we developed a topological model that allows tracing how the information provided by the individual place cells may combine into a large-scale topological map of the navigated space and quantifying the contributions of different neurophysiological parameters. However, previous studies did not include a key physiological aspect–the contribution of the synaptic connections into the processes of assembling the map. Below we will use the topological approach to model how synaptic imperfections can affect the topological structure of the cognitive map, its dynamics and its stability.

The paper is organized as follows. First, we outline the basic ideas and the key concepts used in the topological model–simplexes, simplicial complexes, topological loops, Betti numbers, etc., and explain how these concepts can be applied for describing hippocampal physiology. Second, we outline the parameters of synaptic connectivity and the constructions used to incorporate these parameters into the model. The analyses of the outcomes is given in the Results section and their implications are outlined in the Discussion.

## The Model

### Topological description of the place cell spiking patterns

It is generally believed that the information encoded by the place cell network is represented by the connectivity between the place fields. A specific link is suggested by the classical Alexandrov-Čech’s theorem of Algebraic Topology asserts that the pattern of overlaps between regions that cover a space *X* does, in fact, capture its topological structure^17,18^. The implementation of this theorem is based on constructing the so-called “nerve simplicial complex” ${\mathscr{N}}$, whose vertexes correspond to the individual domains of the cover: one-dimensional (1*D*) links–to their pairwise overlaps, two-dimensional (2*D*) facets–to the triple overlaps and so forth (Fig. 1B). In other words, each *n*^*th*^ order overlap between the place fields is schematically represented by an *n*-dimensional simplex *σ*, so that the full set ${\mathscr{N}}$ of such simplexes incorporates the connectivity structure of the entire place field map^12,19,20^. According to the Alexandrov-Čech’s theorem, this complex has the same “topological shape” as *X*, i.e., the same number of pieces, gaps and holes^21,22^, which provides a link between the place cells’ spiking pattern and the topology of ambient space^12–16^, exploited below.

In general, simplicial complexes provide a convenient framework for describing a wide scope of physiological phenomena. For example, the combinations of the place fields traversed during the rat’s moves correspond to a chain of simplexes Γ = {*σ*_1_, *σ*_2_, …, *σ*_*k*_} that qualitatively represents the shape of the physical trajectory: a closed chain represents a closed physical route, a pair of topologically equivalent chains represent two similar physical paths and so forth^23,24^. The pool of such chains can be used to describe the topological shape of the entire complex–and hence of the corresponding environment. For example, the number of chains that can be deformed into the same vertex defines how many disconnected pieces ${\mathscr{N}}$ has. The number of topologically inequivalent chains that contract to a closed sequence of links defines the number of distinct holes that prevent these chains from contracting to vertexes and so forth^21,22^. In the following, we will refer to these two types of chains, counted up to topological equivalence, as to zero-dimensional (0*D*) and one-dimensional (1*D*) “topological loops” (a standard mathematical terminology), evaluate their numbers–in mathematical terms, zeroth and first Betti numbers, ${b}_{0}({\mathscr{N}})$ and ${b}_{1}({\mathscr{N}})$, and use them to describe shapes of the simplicial complexes.

### Learning dynamics

**Figure 2:**
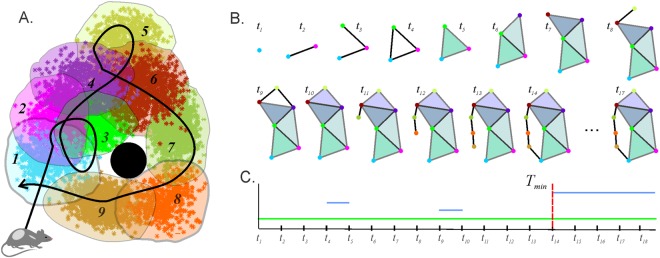
The dynamics of the topological information. (**A**) A mini place field map: nine place fields, enumerated in sequence they are traversed by the animal’s trajectory (black curve). The black circle in the middle represents an obstacle. First the animal enters the place field 1 at a moment, *t*_1_, the nerve complex ${\mathscr{N}}({t}_{1})$ shown on the panel (B) acquires a vertex *σ*_1_ (blue dot). At the time *t*_2_, the animal crosses the domain where the blue and the magenta place fields overlap, and the nerve complex ${\mathscr{N}}({t}_{2})$ acquires the vertex *σ*_2_ and the edge *σ*_12_ between these two vertices. Then the animal enters the place field 3, which contributes a vertex *σ*_3_ and a link *σ*_23_ to ${\mathscr{N}}({t}_{3})$. As the trajectory goes back to the first place field, the complex ${\mathscr{N}}({t}_{4})$ acquires a loop. At the moment *t*_5_ the animal gets into the region where three place fields (1, 2 and 3) overlap; as a result, a two-dimensional simplex *σ*_123_ appears in ${\mathscr{N}}({t}_{5})$ and closes the loop. At time *t*_6_ the animal gets into the intersection of place fields 4, 5 and 6, which contributes the second filled triangle to ${\mathscr{N}}({t}_{6})$, and so on. At the moment *t*_11_ the animal’s trajectory starts to go around the obstacle, and the nerve complex begins to grow a handle which closes into a loop at *t*_14_. After the animal has probed all intersection domains, the structure of the nerve complex ceases to change. (**C**) Each horizontal bar represents the timeline of a 0*D* or a 1*D* loop in ${\mathscr{N}}(t)$. Notice, that there is only one persistent 0*D* loop, because, at all times, there is only one connected piece in ${\mathscr{N}}(t)$. In addition, there are three 1*D* loops: two of them are spurious, appearing at *t*_4_ and at *t*_9_ and disappearing in one time step. In contrast, the loop that appeared at *t*_14_, after all the place fields and their intersections are visited, persists forever and thus represents stable topological information. The time *T*_min_ = *t*_14_ thus provides an estimate for the time required to “learn” this particular map.

To describe how the animal “learns” the environment, one can follow how the nerve complex and its Betti numbers develop in time. In the beginning of exploration, the nerve complex represents connections between the place fields that the animal had time to visit. Such a complex is small and may contain gaps that do not necessarily correspond to physical holes or inaccessible spatial domains of the environment. As the animal continues to navigate, the nerve complex grows and acquires more details; as a result, its the spurious gaps and holes (topological noise) disappear, leaving behind a few *persistent* ones that represent stable topological information (Fig. 2). The minimal time, ${T}_{min}({\mathscr{N}})$, required to recover the correct number of topological loops,

1 $${T}_{{\rm{\min }}}({\mathscr{N}}):\,{b}_{k}({\mathscr{N}},t)={b}_{k}({\mathscr{N}})\,{\rm{for}}\,t > {T}_{{\rm{\min }}}({\mathscr{N}})\,{\rm{and}}\,k\ge 0,$$

can be used as a theoretical estimate of the time needed to learn path connectivity^12^. In the case of the environment illustrated on Fig. 1A, with the Betti numbers ${b}_{0}( {\mathcal E} )={b}_{1}( {\mathcal E} )=1$, ${b}_{k > 1}( {\mathcal E} )=0$, the nerve complex is expected to have the same “topological barcode”: ${b}_{0}({\mathscr{N}},t > {T}_{{\rm{\min }}})={b}_{1}({\mathscr{N}},t > {T}_{{\rm{\min }}})=1$, ${b}_{k > 1}({\mathscr{N}},t > {T}_{{\rm{\min }}})=0$.

### Temporal coactivity complex

From the physiological perspective, the arguments based on the analyses of place fields and trajectories provide only an indirect description of information processing in the brain. In reality, the hippocampus and the downstream brain regions do not have access to the shapes and the locations of the place fields or to other artificial geometric constructs used by experimentalists to visualize their data. Physiologically, the information is represented via neuronal spiking activity: if the animal enters a location where several place fields overlap, then there is a *probability*, modulated by the rat’s location, that the corresponding place cells will produce spike trains that overlap *temporally*. This pattern of coactivity signals to the downstream brain areas that the regions encoded by these place cells overlap. Thus, in order to describe the learning process in proper terms, one needs to construct a *temporal* analogue of the nerve complex based only on the spiking signals, which is, in fact, straightforward. Indeed, one can represent an active place cell, *c*_*i*_, by a vertex *v*_*i*_; a pair of coactive place cells, *c*_*i*_ and *c*_*j*_—by a bond *σ*_*ij*_ between the vertices *v*_*i*_ and *v*_*j*_; a coactive triple of place cells, *c*_*i*_, *c*_*j*_ and *c*_*k*_—by a three vertex simplex *σ*_*ijk*_ and so on^12,19,20^. This construction produces a time-dependent “coactivity complex” ${\mathscr{T}}(t)$—a temporal analogue of the nerve complex ${\mathscr{N}}(t)$ constructed above, whose dynamics can also be used to model topological learning, e.g., to compute the learning time from the spiking data, ${T}_{{\rm{\min }}}({\mathscr{T}}\,)$, and so forth^12^.

### Cell assembly complex

The construction of a temporal complex can be refined to reflect more subtle physiological details, e.g., the functional organization of the hippocampal network. Studies of place cells’ spiking times point out that these neurons tend to fire in “assemblies”—functionally interconnected groups that are believed to synaptically drive a population of “readout” neurons in the downstream networks^25–29^. The latter are wired to integrate spiking inputs from their respective cell assemblies and actualize the connectivity relationships between the regions encoded by the corresponding place cells^29,30^.

**Figure 3:**
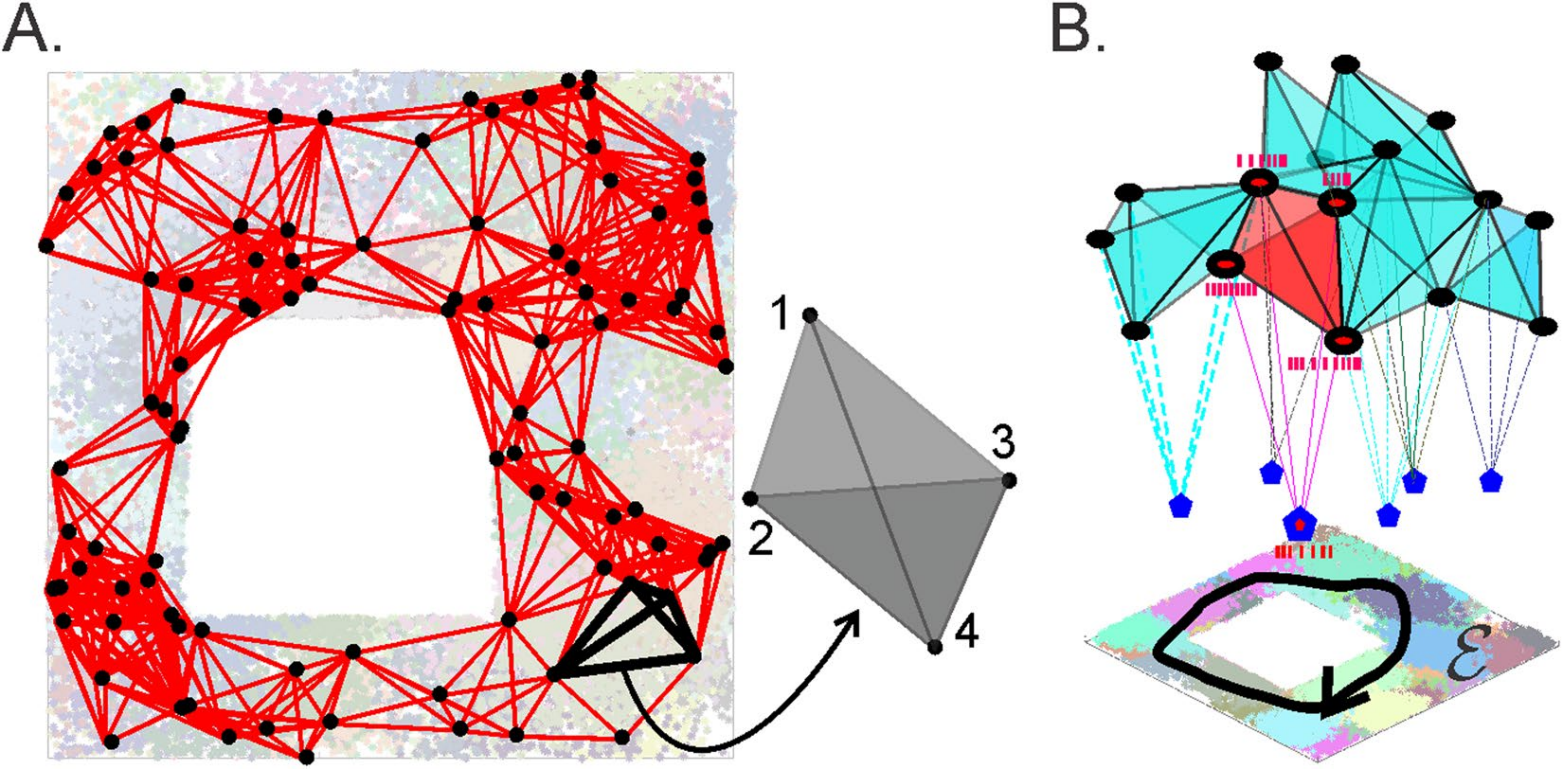
Coactivity graphs and cell assembly complexes. (**A**) Active place cells are represented by the vertexes of the coactivity graph ${\mathscr{G}}$ (black dots placed at the centers of the corresponding place fields). Two vertexes are connected by an edge if the corresponding place cells exhibit coactivity. The fully connected subgraphs of ${\mathscr{G}}$–its cliques, e.g., the four interconnected black links on the right–correspond to the cell assemblies. (**B**) The collection of cliques viewed as simplexes of the cell assembly complex ${{\mathscr{T}}}_{CA}$, represent the topology of underlying environment. In the model, every place cell in an assembly is synaptically connected to a readout neuron (blue pentagons). The spikes from an active place cells *c*_*k*_ (an ignited cell assembly *ς* is shown in red) transmit to a readout neuron with probability ${p}_{k} < 1$ and the readout neuron responds with probability ${q}_{\varsigma } < 1$.

This structure can be represented by the cell assembly complex, ${{\mathscr{T}}}_{CA}$—a temporal coactivity complex whose maximal simplexes represent cell assemblies, rather than arbitrary combinations of coactive place cells. A convenient implementation of this construction is based on the classical “cognitive graph” model, in which place cells *c*_*i*_ are represented as vertexes *v*_*i*_ of a graph ${\mathscr{G}}$, while the connections (functional or physiological) between pairs of coactive cells are represented by the links, *σ*_*ij*_ = [*v*_*i*_, *v*_*j*_] of ${\mathscr{G}}$^30–32^. The place cell assemblies *σ* = [*c*_1_, *c*_2_, …, *c*_*n*_] then correspond to fully interconnected subgraphs of ${\mathscr{G}}$, i.e., to its maximal cliques^15,16^. Since a clique *σ*, as a combinatorial object, can be viewed as a simplex span by the same sets of vertexes, the collection of cliques of the coactivity graph ${\mathscr{G}}$ produces a so-called clique simplicial complex^33^, which represents the population of place cell assemblies and may hence be viewed as a cell assembly complex ${{\mathscr{T}}}_{CA}$ (Fig. 3).

### Phenomenological description of the synaptic parameters

In the previous studies, we demonstrated that such complexes can acquire a correct topological shape in a biologically plausible period of time, in both planar and in voluminous environments, provided that the simulated spiking parameters values fall into the biological range^12–16^. However, the organization and the dynamics of these complexes did not reflect the parameters of synaptic connectivity, e.g., the mechanisms of transferring, detecting and interpreting neuronal (co)activity in the hippocampus and in the downstream networks. To account for these components, the topological model requires a basic modification: a particular coactivity pattern should be incorporated into an *effective* coactivity complex ${{\mathscr{T}}}_{{\rm{eff}}}$ not by the virtue of being merely produced, but by the virtue of being produced, transmitted and ultimately detected by a readout neuron. In other words, only *detected* activity of a place cell *c*_*i*_ should be represented by a vertex *v*_*i*_; a *detected* coactivity of two place cells, *c*_*i*_ and *c*_*j*_—by a bond *σ*_*ij*_, a *detected* coactivity of three place cells, *c*_*i*_, *c*_*j*_ and *c*_*k*_—by a simplex *σ*_*ijk*_ and so on (Fig. 1B). The resulting complex ${{\mathscr{T}}}_{{\rm{eff}}}$ then constitutes a basic phenomenological model of a cognitive map assembled from the spiking inputs transmitted through imperfect synaptic connections.

### Statistical approach

The mechanisms of spike generation, transmission and detection are probabilistic in nature. Transmitting action potentials requires producing a sufficient number of synaptic contacts in suitable locations of the postsynaptic neuron’s membrane, releasing proper amount of neurotransmitter at each synapse at suitable times, inducing the excitatory postsynaptic potential (EPSP) of required magnitudes, etc., all of which involve probabilistic mechanisms^34,35^. In addition, there may appear flaws and glitches in the axons, synaptic clefts and in the structure of the postsynaptic membrane’s polarization. Thus, there exists a probability ${p}_{k} < 1$ that a *k*^th^ connection in a cell assembly *ς* will induce sufficient EPSP in the readout neuron’s membrane and a probability ${q}_{\varsigma } < 1$ that the latter will spike upon receiving the inputs (Fig. 3C).

In principle, these values could be estimated from the synaptic configuration of each individual assembly, which, however, would present a tremendous computational challenge^36–38^. In order to avoid such complications, we will assume a basic statistical approach. First, we will regard the probabilities *p*_*k*_, and *q*_*ς*_ as the prime parameters that describe the synaptic connections with the readout neuron. Second, we will view *p*_*k*_ and *q*_*ς*_ as random variables, distributed according to a unimodal distribution, $P(p|\hat{p},{{\rm{\Delta }}}_{p})$ and $Q(q|\hat{q},{{\rm{\Delta }}}_{q})$ were $\hat{p}$ and $\hat{q}$ are the modes (the characteristic values) and Δ_*p*_ and Δ_*q*_ define the corresponding variances. Third, we will disregard synaptic plasticity processes and assume that the distributions are stationary, i.e., that the modes and the variances are fixed. Fourth, we will assume that both variables are distributed lognormally, as suggested by experimental observations^39–41^. We will also define the variances as functions of the modes, ${{\rm{\Delta }}}_{p}\propto {\hat{p}}^{2}$ and ${{\rm{\Delta }}}_{q}\propto {\hat{q}}^{2}$, which will allow us to exclude non-biological statistics and to study the topological properties of the emerging cognitive maps as functions of just two parameters, $\hat{p}$ and $\hat{q}$.

### Implementation

In order to isolate the effects of varying transition probabilities while keeping the temporal structure of the presynaptic spike trains “clamped”, we use the spiking data that was precomputed for the “ideal” synaptic connections (*p*_*k*_ = *q*_*ς*_ = 1), and then screen out some of the spikes, to match each individual transmission probabilities ${p}_{k} < 1$ and to simulate the readout neurons’ responses to the igniting cell assemblies with probabilities ${q}_{\varsigma } < 1$.

To evaluate the latter, we reasoned as follows. Since in our approach the cell assemblies are modeled as the cliques of the coactivity graph ${\mathscr{G}}$, i.e., as composite objects assembled from *n*(*n* − 1)/2 pairs of place cells, the probabilities of igniting the higher order place cell combinations can be computed from the pairwise coactivities. Indeed, if the spikes produced by the place cells *c*_*i*_ and *c*_*j*_ are transmitted to the readout neuron with the probabilities *p*_*i*_ and *p*_*j*_ respectively, then the corresponding pairwise coactivity occurs with the probability *p*_*i* _*p*_*j*_. The probability of a third order coactivity, e.g., the ignition of a clique *σ*_*ijk*_ = [*c*_*i*_, *c*_*j*_, *c*_*k*_] is then defined by the probability of transmitting the coactive pairs *σ*_*ij*_ = [*c*_*i*_, *c*_*j*_], *σ*_*jk*_ = [*c*_*j*_, *c*_*k*_], and *σ*_*ik*_ = [*c*_*i*_, *c*_*k*_] and detecting the result with the probability *q*_*ς*_; the probability of igniting the fourth order cliques is defined by the corresponding six coactive pairs and so forth.

With these assumptions, one can test how the spike transmission and detection probabilities affect the emergence of a spatial map, e.g., how synaptic depletion affects spatial learning, how the learning times and the topological structure of the cognitive map depend upon the strengths of synaptic connections between the place cells and the readout neurons, at what point spatial learning may fail, and so on.

## Results

### Learning times

**Figure 4:**
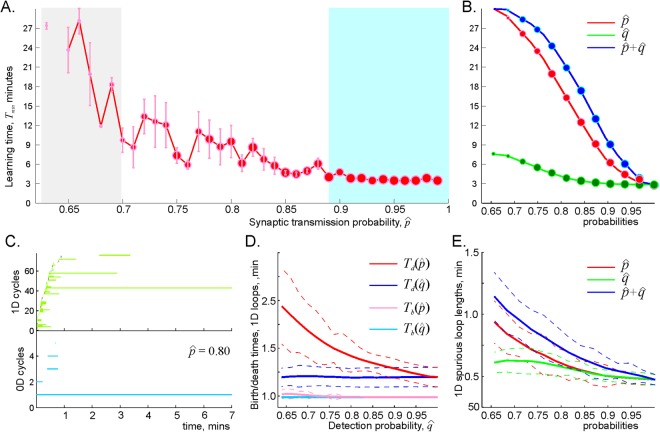
Synaptic transmission probability and the learning times. (**A**) The dependence of the learning time *T*_min_ on the ensemble mean spike transmission probability, $\hat{p}$, in an ensemble of *N* = 400 neurons with a mean firing rate of *f* = 28 Hz, and mean place field size 30 cm. The learning times, *T*_min_, are computed for 40 values of $\hat{p}$, ranging between $\hat{p}=1$ and $\hat{p}=0.6$. The size of the data points represents the percentage of the outcomes with the correct Betti numbers (${b}_{0}({{\mathscr{T}}}_{eff})={b}_{1}({{\mathscr{T}}}_{eff})=1$). For high probability of spike transmissions ($\hat{p} > \mathrm{90 \% }$, blue-shaded area) the learning time remains nearly unchanged; as $\hat{p}$ drops further, the learning time increases at a power rate. As the transmission probability approaches the critical value ${\hat{p}}_{{\rm{crit}}}$ (in this case, ${\hat{p}}_{{\rm{crit}}}\approx 0.64$, gray-shaded area), the learning times become large and highly variable; below ${\hat{p}}_{{\rm{crit}}}$ the place cell ensemble fails to form the correct topological map, even though the place cells exhibit perfectly functional, spatially specific firing pattern. (**B**) The effect produced by the decreasing postsynaptic response probability ($\hat{q}$, green curve) is similar, but smaller than the effect produced by the decreasing spike transmission probability ($\hat{p}$, red curve). The combined effect (blue curve) is approximately additive, dominated by $\hat{p}$-dependence. (**C**) Timelines of 0*D* (blue) and 1*D* (green) topological loops computed for the same map and $\hat{p}=0.8$. This panel serves as an illustration for the next two panels. (**D**) On average, the spurious loops appear in about a minute after the onset of the navigation, which approximately corresponds to the time required to run around the central hole of the environment (Fig. 1A). As the probabilities $\hat{p}$ or $\hat{q}$ decrease, the birth times (${T}_{b}(\hat{p})$ and ${T}_{b}(\hat{q})$ the pink and the light blue curve correspondingly) do not change significantly. In contrast, the times required by the spurious loops to disappear grow significantly: ${T}_{d}(\hat{p})$ (red curve) grows by over 100%, and ${T}_{d}(\hat{q})$ (blue curve) increases by a few percent. (**E**) The dependence of the spurious loops’ length as a function of spike transmission $\hat{p}$, and the readout neurons’ response probability, $\hat{q}$.

Lowering the characteristic probability of spike transmissions and the characteristic probability of the readout neurons’ responses produces an uneven delay in spatial learning times (Fig. 4A). If the spike transmission probability is high (typically $0.9\le \hat{p}\le 1$), then the small variations of $\hat{p}$ do not inflict a strong impact on *T*_min_, i.e., the time required to learn the spatial map in a network with strong synaptic connections is nearly unaffected by occasional omissions of spikes. On the other hand, as $\hat{p}$ lowers to a certain critical value ${\hat{p}}_{{\rm{crit}}}$, the learning times become high and, as $\hat{p}$ drops below ${\hat{p}}_{{\rm{crit}}}$, the coactivity complex fails to produce the correct topological shape of the environment in finite time. For the intermediate values, the learning time increases at a power rate,

2 $${T}_{{\rm{\min }}}\propto {(\hat{p}-{\hat{p}}_{{\rm{crit}}})}^{-\kappa },$$

where *κ* ranges between 0.1 and 0.5 for different values of *s*, *f*, *N*. The effects produced by the diminishing probability of the postsynaptic neurons’ responses, $\hat{q}$, are qualitatively similar but weaker than the effects of lowering the spike transmission probability $\hat{p}$: the learning time shows a weak or no dependence for large $\hat{q}$ (typically $0.8\le \hat{q}\le 1$), followed by the power divergence near the critical value,

3 $${T}_{min}\propto {(\hat{q}-{\hat{q}}_{{\rm{c}}{\rm{r}}{\rm{i}}{\rm{t}}})}^{-\varkappa },$$

with a small power exponent $\varkappa \approx 0.1$ (Fig. 4B). Lowering both $\hat{p}$ and $\hat{q}$ simultaneously leads to a combined, accelerated increase of the learning time (Fig. 4B).

An implication of this phenomenon is that, $\hat{p}$ and $\hat{q}$, being independent characteristics of synaptic efficacy, can also compensate for each other’s alterations: the effect of decreasing $\hat{q}$ can be counterbalanced by increasing $\hat{p}$ and vice versa. Indeed, the dependencies (2) and (3) also define the changes of the learning time induced by small variations in the transmission probability,

4 $${\delta }_{p}{T}_{{\rm{\min }}}\propto -\,\frac{\kappa \,{T}_{{\rm{\min }}}}{\hat{p}-{\hat{p}}_{{\rm{crit}}}}\delta \hat{p},$$

and by the variations of the postsynaptic neuron’s response probability,

5 $${\delta }_{q}{T}_{min}\propto -\,\frac{\varkappa {T}_{min}}{\hat{q}-{\hat{q}}_{{\rm{c}}{\rm{r}}{\rm{i}}{\rm{t}}}}\delta \hat{q}.$$

These relationships imply that the compensation of the changes of the learning time, *δ*_*p*_*T*_min_ = −*δ*_*p*_*T*_min_, is achieved if

6 $$\frac{\kappa \,\delta \hat{p}}{\hat{p}-{\hat{p}}_{{\rm{c}}{\rm{r}}{\rm{i}}{\rm{t}}}}\approx -\,\frac{\varkappa \,\delta \hat{q}}{\hat{q}-{\hat{q}}_{{\rm{c}}{\rm{r}}{\rm{i}}{\rm{t}}}}.$$

Notice, that this dependence is *T*_min_-independent and nonlinear: given a particular value of $\delta \hat{p}$, the required compensatory change of $\delta \hat{p}$ depends on the initial values of both $\hat{p}$ and $\hat{q}$.

### Dynamics of the effective coactivity complex

The failures of the learning and memory capacity caused by deterioration of synapses are broadly discussed in the literature^42–44^. However, empirical observations provide only correlative links between these two scopes of phenomena. Indeed, the direct effects of the synaptic changes, e.g. the alterations of EPSP magnitudes, the spike transmission probabilities, the parameters of synaptic plasticity, etc., occur at cellular scale. It therefore remains unclear how such changes may accumulate at the network scale to control the net structure and the dynamics of the large-scale memory framework at the organismal level. The topological model allows addressing these questions at a phenomenological level, in terms of the structure of the coactivity complex ${{\mathscr{T}}}_{{\rm{eff}}}$—its topological shape, its size, the dynamics of its topological loops and so forth, in response to the changes of synaptic parameters.

For example, one can evaluate the statistics of birth (*T*_*b*_) and death (*T*_*d*_) times of the topological loops in the coactivity complex. As shown on (Fig. 4C), the time when spurious loops begin to emerge depend only marginally on spike transmission probability. However, the spurious loops’ disappearance times are impacted much stronger: although *T*_*d*_ shows only weak $\hat{p}$-dependence at high $\hat{p}$, further suppression of the spike transmissions may double or triple the loops’ disappearance time. The contribution of the decreasing response probability $\hat{q}$ is similar, but at a smaller scale: over the range ${\hat{q}}_{{\rm{crit}}} < \hat{q}\le 1$, the learning time changes only by a few percent (Fig. 4D). Similar effects are indicated by the $\hat{p}$- and $\hat{q}$-dependencies of the spurious loops’ lengths, which may grow significantly as a result of the diminishing spike transmission probability, but increase only by 30–50% due to the lowering probability of the readout neuron’s responses (Fig. 4E).

Taken together, these results explicate the power growth of the learning times indicated by (2) and (3) and provide a simple intuitive explanation for the decelerated spatial learning and its eventual failure caused by the synaptic depletion: according to the model, lowering synaptic efficacy stabilizes spurious topological loops in the coactivity complex, making it harder to extract physical information from the transient noise.

**Figure 5:**
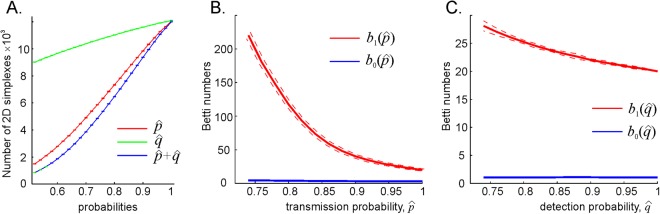
Deterioration of the coactivity complex. (**A**) The size of the complex shrinks with the diminishing spike transmission ($\hat{p}$-dependence, red line) and the readout neurons’ response ($\hat{q}$-dependence, green line) probability at a power rate, ${N}_{2}\propto {(\hat{p}-{\hat{p}}_{crit})}^{\delta }$, and ${N}_{2}\propto {(\hat{q}-{\hat{q}}_{crit})}^{\varepsilon }$. In this case, $\delta \sim 1.5$ and $\varepsilon \sim 0.4$. The combined effect of reducing both $\hat{p}$ and $\hat{q}$ is illustrated by the blue line. (**B**) The spurious topological loops in ${{\mathscr{T}}}_{{\rm{eff}}}$ proliferate exponentially with decreasing transmission probability $\hat{p}$. The blue and the red curve show the dependence of zeroth and first Betti numbers on the transmission probability, ${b}_{0}(\hat{p})$ and ${b}_{1}(\hat{p})$ respectively. (**C**) The dependence of the numbers of 0*D* and 1*D* on the readout neuron’s response probability $\hat{q}$ is weaker: while ${b}_{1}(\hat{q})$ exhibits a moderate growth, the ${b}_{0}(\hat{q})$ remains unchanged.

Additional perspective on the mechanisms of the cognitive map’s deterioration is produced by analyzing the size of the coactivity complex and the number of the topological loops in it. As shown on Fig. 5A, the decay of $\hat{p}$ causes rapid decay of the coactivity complex’s size: the number of its two-dimensional simplexes (i.e., links in the coactivity graph, see below) drops as ${N}_{2}\propto {(\hat{p}-{\hat{p}}_{crit})}^{\delta }$, where $\delta  > 1$. Diminishing $\hat{q}$ also shrinks the coactivity complex, but at a slower rate, ${N}_{2}\propto {(\hat{q}-{\hat{q}}_{crit})}^{\varepsilon }$ with $0 < \varepsilon  < 1$. However, despite the shrinking size of the coactivity complex, the number of 1*D* spurious loops in it grows exponentially, $\mathrm{log}({b}_{1})\propto ({\hat{p}}_{crit}-\hat{p})$, from a few dozen to a few hundred, accompanied by a weak ${b}_{0}(\hat{p})$ increase (Fig. 5B). Similar effects are produced by the lowering detection probability, but again, at a much smaller scale: the number of 1*D* loops, ${b}_{1}(\hat{q})$, increases by about 30% while the ${b}_{1}(\hat{q})$ does not change (Fig. 5C).

These outcomes indicate that, as a result of weakening synaptic connections, the spurious topological loops do not only stabilize but also proliferate, thus preventing the effective coactivity complex from capturing the correct topology of the ambient space. In physiological terms, the model predicts that weakening synapses produce large numbers of longer-lasting topological defects in the cognitive map, which results in a rapid increase of the time required to learn the topology of the physical environment from poorly communicated spiking inputs.

### Critical probabilities

As indicated above, if the synaptic efficacies are too weak, i.e., if either the spike transmission or the postsynaptic response probability drops below their respective critical values, then the effective coactivity complex ${{\mathscr{T}}}_{{\rm{eff}}}$ may disintegrate into a few disconnected pieces and lose its physical shape–a single large piece with a hole in the middle (${b}_{0}({\mathscr{N}})={b}_{0}( {\mathcal E} )$ and ${b}_{1}({\mathscr{N}})={b}_{1}( {\mathcal E} )$, Fig. 1C), may be replaced by a “spongy” configuration containing several smaller pieces with many holes^45,46^. Thus, the cognitive map may appear in two distinct states: for $\hat{p} > {\hat{p}}_{{\rm{crit}}}$ and $\hat{q} > {\hat{q}}_{{\rm{crit}}}$ the spurious topological defects can be separated from the topological signatures of the physical environment, whereas below the critical values, topological noise overwhelms physical information. The transition between these two states is accompanied an increased variability of the learning times (Fig. 4A) and by their power divergence caused by the exponential proliferation of the topological fluctuations in the coactivity complex. These effects suggest that, near ${\hat{p}}_{{\rm{crit}}}$ and ${\hat{q}}_{{\rm{crit}}}$, the coactivity complex may experience a phase-like transition^47–49^ from a regular state, in which spatial learning is effective to an irregular state, in which spatial learning fails.

Since in most of the studied cases, the critical synaptic transmission probability, ${\hat{p}}_{{\rm{crit}}}$, is easier to achieve than the critical probability of the readout neuron’s responses, ${\hat{q}}_{{\rm{crit}}}$, we studied the dependence of the former on the ensemble parameters, i.e., on the number of place cells in the ensemble, their mean firing rate and the mean place field size,

7 $${\hat{p}}_{{\rm{crit}}}={\hat{p}}_{{\rm{crit}}}(s,f,N).$$

**Figure 6:**
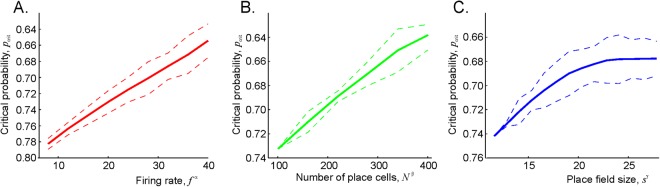
Critical transmission probability as a function of spiking parameters. (**A**) Increasing the mean ensemble firing rate *f* reduces the critical transmission probability at a superlinear rate. Shown is the dependence ${\hat{p}}_{{\rm{crit}}}$ vs. *f*^−*α*^, where *α* ≈ 4. (**B**) As the number *N* of active place cell in the ensemble grows, the critical transmission probability drops as ${\hat{p}}_{{\rm{crit}}}\propto {N}^{\beta }$, with *β* ≈ 1.2. (**C**) The transmission probability also drops as a function of the mean place field size *s*, ${\hat{p}}_{{\rm{crit}}}\propto {s}^{\gamma }$, *γ* ≈ 0.4, as long as the place fields are not too large. As the place cells loose spatial specificity of firing activity (*s* > 20), a low transmission probability cannot be sustained.

The results shown on Fig. 6 reveal power-law dependencies: ${\hat{p}}_{{\rm{crit}}}\propto {f}^{\alpha }$, ${\hat{p}}_{{\rm{crit}}}\propto {N}^{\beta }$, and a more complex *s*-dependence. Since the domain of these dependences covers the experimentally observed range of parameters, the results can be interpreted physiologically. First, if the ensemble firing rates are too low, or if the place fields are too meager, or the number of the active neurons is too small (the left ends of the dependencies shown on Fig. 6), then the corresponding place cell ensemble fails to learn the spatial map of the environment, even if the synaptic connections are nearly perfect ($\hat{p} > 0.75$), which corresponds to the results discussed in^12–14^. As the mean firing rate and the number of active neurons increase, the critical probability ${\hat{p}}_{{\rm{crit}}}$ steadily *decreases*, which implies that the synaptic depletion may be compensated by enhancing neuronal activity, as observed in experimental studies^50–52^. In contrast, the dependence ${\hat{p}}_{{\rm{crit}}}(s)$ saturates and even reverses its direction for overly large place fields. This, however, is a natural result since poor spatial specificity of the place cells’ spiking should prevent successful spatial leaning even for large $\hat{p}$^12–14^.

Electrophysiological studies show that only up to 10−20% of spikes are transmitted between the neurons in CA1 slices, which is lower than the critical values discussed above^39,53,54^. However, the results shown on Fig. 6C imply that the experimentally observed values of $\hat{p}$ can be achieved for larger values of *N*, i.e., in larger place cell ensembles. Interpolated ${\hat{p}}_{{\rm{crit}}}(N)$ dependence indicates that the physiological values of ${\hat{p}}_{crit}\sim 0.1-0.2$, can be achieved for the ensembles of N ~ 3000 cells, which corresponds to the experimentally observed values^55,56^.

### Learning region

One of the key characteristics of the place cell spiking activity produced by the topological model is the range of the spiking parameters, for which the coactivity complex can assume a correct topological shape in a biologically feasible period. Geometrically, this set of parameters forms a domain in the parameter space that we refer to as the *learning region*, ${\mathcal L}$^12^. The shape and the size of the learning region varies with the geometric complexity of the environment and the difficulty of the task: the simpler is the environment and easier the task, the larger is ${\mathcal L}$, i.e., the wider the range of physiological values that permits learning a map of that space^57,58^. On the other hand, a larger ${\mathcal L}$ implies a greater range within which the brain can compensate for physiological variation: if one parameter begins to drive the system outside the learning region, then successful spatial learning can still occur, provided that compensatory changes of other parameters can keep the neuronal ensemble inside ${\mathcal L}$. For example, a reduction of the number of active neurons can sometimes be compensated by adjusting the firing rate or the place field size in such a way as to bring their behavior back within the perimeter of the learning region.

Interpreting the parameters of a given place cell ensemble in the context of its placement within or relative to the learning region sheds light on the mechanism of memory failure caused by certain neurophysiological conditions, e.g., by the Alzheimer Disease^59,60^, or by aging^61,62^ or certain chemicals, e.g., ethanol^63,64^, cannabinoids^65,66^ or methamphetamines^67,68^, which appear to disrupt spatial learning by gradually shifting the parameters of spiking activity beyond the learning region. On the other hand, the performance of a place cell ensemble can improve by enhancing place cells spiking activity pharmacologically or by Deep Brain Stimulation^69^, or by modulating the hippocampal neural oscillations^70^, as the model predicts^12–14^.

**Figure 7:**
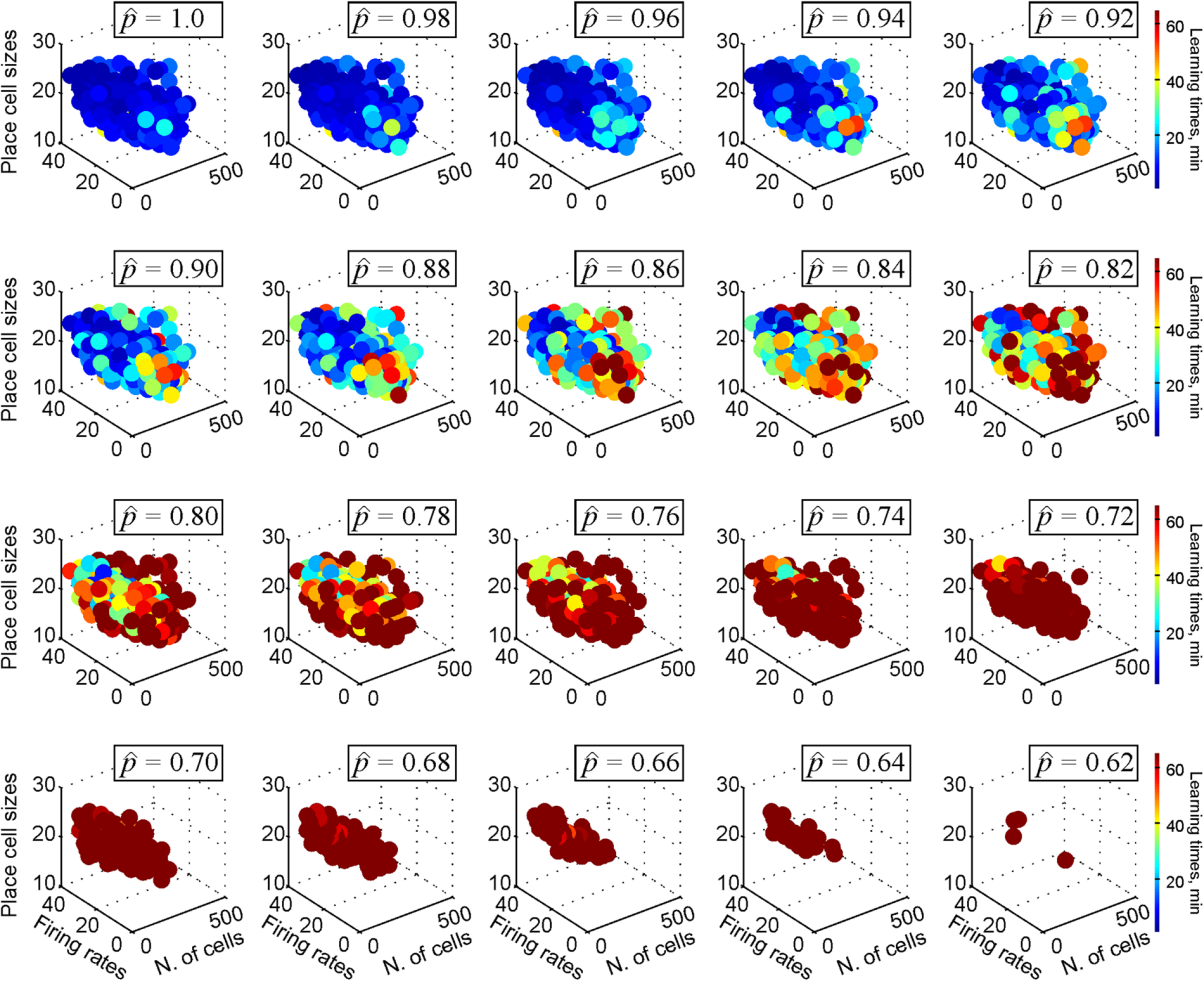
Synaptic connection strengths affect spatial learning. By simulating spatial learning in a given environment for various ensemble-mean values of place field size (4 < *s* < 30), firing rates (4 < *f* < 40) and the size of the place cell population (50 < *N* < 500), we can estimate the domain within a large parametric space representing the set of place cell ensembles that can produce a correct spatial map–the *Learning Region* (${\mathcal L}$). Each dot represents a hippocampal state as defined by a particular triple (*s*, *f*, *N*); the color of the dot is the mean time required for a given ensemble to encode an accurate map of the environment’s features, averaged over ten place field configurations. Outside ${\mathcal L}$ learning is inaccurate or unreliable. As the transmission probability $\hat{p}$ decreases, the learning region shrinks and disappears as the transmission probability $\hat{p}$ approaches ${\hat{p}}_{{\rm{crit}}}$.

In contrast, diminishing spike transmission probability produces a qualitatively different effect: as shown on Fig. 7, it reduces the learning region from its original (largest) size at $\hat{p}=1$ to its compete disappearance at the critical value $\hat{p}={\hat{p}}_{{\rm{crit}}}$. During this process, the time required to form the cognitive map of the environment progressively increases from a few minutes to over an hour (Fig. 7).

Physiologically, these results suggest that if the synaptic connections are too weak, then the system may fail to form a map not only because the parameters of neuronal firing are pushed beyond a certain “working range,” but also because that range itself may diminish or cease to exist. In particular, the fact that the learning region disappears if the transmission probability drops below the critical value, implies that the deterioration of memory capacity caused by synaptic failure may not be compensated by increasing the place field’s firing rates or by recruiting a larger population of active neurons, i.e., some neuropathological conditions may indeed be primarily “synaptic” in nature^42^.

### Deteriorating cognitive graph

A simple alternative explanation of these results can be provided in terms of the place cell coactivity statistics. As pointed out in Section 2, the collection of the unique pairs of the coactive place cells in a network with ideal synaptic connections ($\hat{p}=\hat{q}=1$) is represented by the coactivity graph ${\mathscr{G}}$. The imperfect synapses diminish the pool of the transmitted and the detected coactive pairs, which then corresponds to a smaller, *effective* coactivity graph ${{\mathscr{G}}}_{{\rm{eff}}}(\hat{p})\subset {\mathscr{G}}$. The corresponding set of higher order coactivities–the effective coactivity complex ${{\mathscr{T}}}_{eff}(\hat{p})$ induced from ${{\mathscr{G}}}_{{\rm{eff}}}(\hat{p})$ is a subcomplex of the original coactivity complex, with potentially altered topological properties. The net results discussed above imply that, for high transmission probabilities, the effective coactivity complex ${{\mathscr{T}}}_{eff}$ retains the original topological shape of ${\mathscr{T}}$, but as $\hat{p}$ diminishes, the effective complex shrinks, acquires multiple topological defects and eventually loses its correct shape, indicating a failure of spatial learning.

An illuminating perspective on the changing structure of the coactivity graph ${\mathscr{G}}$ described above is provided by its Forman curvature–a combinatorial analogue of the standard differential-geometric notion of curvature^71,72^. The Forman curvature is adopted for discrete, combinatorial structures, such as datasets, networks and graphs^73–76^, and can be flexibly defined in terms of an individual network’s characteristics–the “weights” of its vertexes and edges. Specifically, for an undirected edge *e* with a weight *w*(*e*) connecting the vertexes *v*_1_ and *v*_2_ with the weights *w*(*v*_1_) and *w*(*v*_2_) it is defined as

8 $${R}_{F}(e)=w({v}_{1})+w({v}_{2})-\sum _{{e}_{{v}_{1},{v}_{2}}}(w({v}_{1})\sqrt{\frac{w(e)}{w({e}_{{v}_{1}})}}+w({v}_{2})\sqrt{\frac{w(e)}{w({e}_{{v}_{2}})}}),$$

where the summation goes over the other edges ${e}_{{v}_{1}}$ and ${e}_{{v}_{2}}$ connecting to *v*_1_ and *v*_2_. The curvature associated with a vertex, *R*_*F*_(*v*), equals to the mean curvature of the edges that meet at *v*.

**Figure 8:**
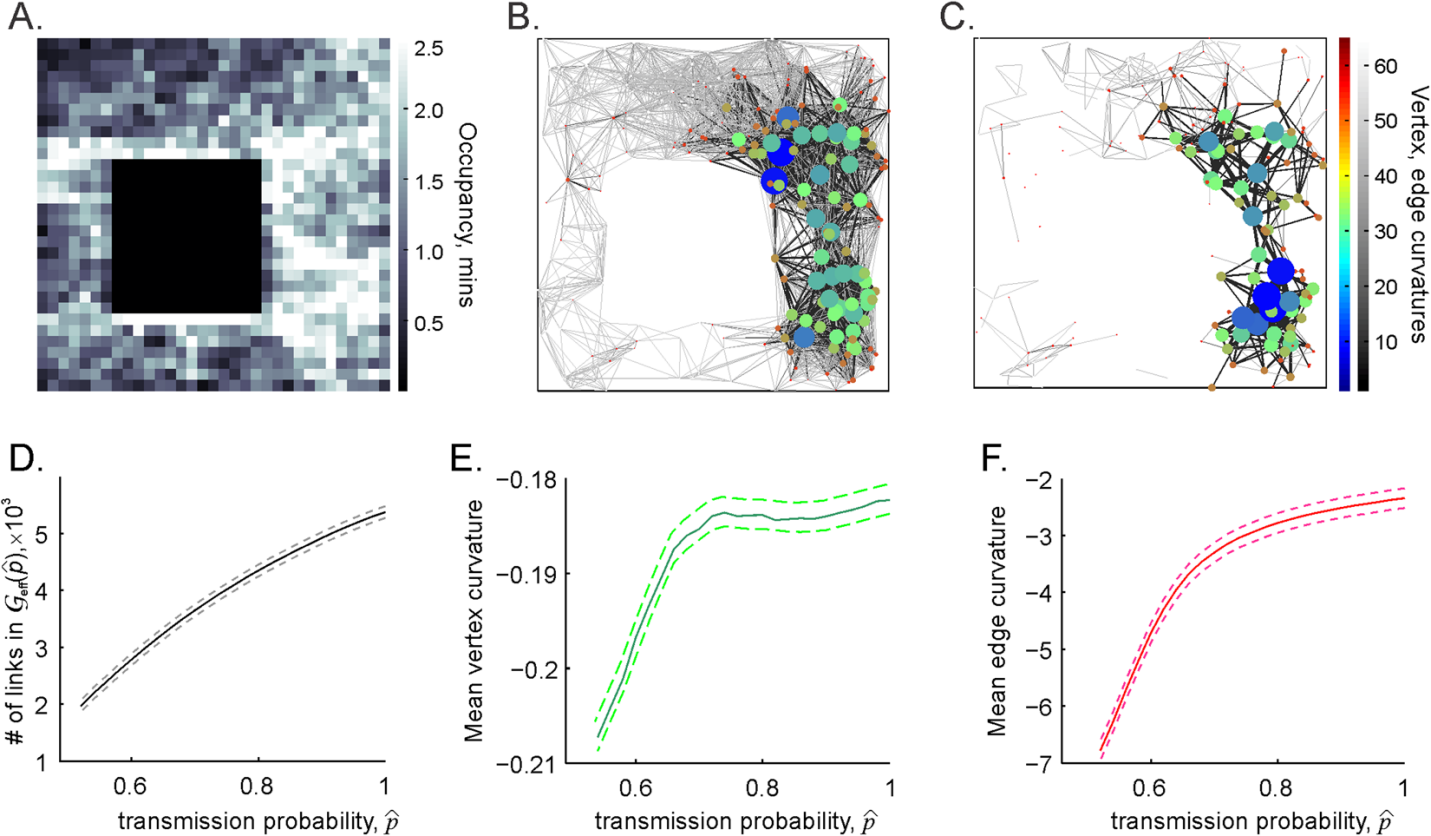
The decay of the coactivity graph. (**A**) The occupancy map of the simulated rat’s trajectory. The highlighted areas indicate where the rat spends more time (gray colorbar). (**B**) The coactivity graph ${\mathscr{G}}$ computed for perfect connections ($\hat{p}=\hat{q}=1$): the thickness and the shade of the edges (gray colorbar), as well as the sizes and the colors of the vertices (jet colorbar) are scaled according to their respective Forman curvatures. (**C**) The effective coactivity graph ${{\mathscr{G}}}_{{\rm{eff}}}$ computed for the spike transmission probability $\hat{p}=0.65$ is significantly sparser that at $\hat{p}=1$: only about 10% of edges with high Forman curvatures remain. Notice that in both cases, the edges and vertexes with high curvature concentrate in the area that were visited most by the rat (panel A). (**D**) The effective coactivity graph shrinks as a function of the transmission probability decay. The mean Forman curvature of the vertexes (panel E) and of the edges (**F**) also decreases as a function of decaying $\hat{p}$, as the low-curvature vertexes and edges disappear.

As discussed in^73–76^, the values *R*_*F*_(*e*) and *R*_*F*_(*v*) provide a measure of the divergence of information flow across the network, highlighting the most “important” edges and vertexes. Applying these ideas to the case of the coactivity graph, weighing its vertexes with the number of spikes produced by the corresponding place cells and its edges with correlation coefficients between the corresponding pairs of cells, reveals that the distribution of the resulting Forman curvatures follows the structure of the occupancy map (Fig. 8A,B). In other words, the most visited vertexes and edges appear as the most “curved” ones, controlling the flow of information in ${{\mathscr{G}}}_{{\rm{eff}}}$.

This quantification also allows a natural interpretation of the effective coactivity graph’s dynamics: as the spike transmission probability $\hat{p}$ decreases, ${{\mathscr{G}}}_{{\rm{eff}}}(\hat{p})$ sheds the “least important” vertexes and links with low curvatures (Fig. 8C,D,E). Thus, as the synaptic efficacies weaken, the emerging effective coactivity graph reflects only the most persistently firing place cells and the highly correlated pairs of such cells, which can sustain the full topological connectivity information, but only for so long. As the synapses deteriorate below critical value, $\hat{p} < {\hat{p}}_{{\rm{crit}}}$, the corresponding effective coactivity complex acquires an irreparable amount of topological defects and fails to encode the correct topological map of the environment.

## Discussion

Countless observations point out that deteriorations of synapses often accompany memory deficiencies. For example, the recurrent connectivity of CA3 area of the hippocampus and the many-to-one projections from the CA3 to the CA1 area^29,77^ suggest that the CA1 cells may provide readouts for the activity of the CA3 place cell assemblies^16^. Behavioral and cognitive experiments demonstrate that weakening of the synapses between these two areas, a reduction in the number of active neurons in either domain, diminishing neuronal activity and so forth, correlate with learning and memory deficiencies observed, e.g., in Alzheimer’s disease^59,60^ or in aging subjects^61,62^. However, without a theoretical framework that can link the “synaptic” and the “organismal” scales, the detailed connections between these two scopes of phenomena are hard to trace. For example, if the spike transmission rate in an ensemble of place cells decreases, e.g., by 5%, will the time required to learn the environment increase by 1%, 5% or by 50%? Does the outcome depend on the “base” level of the transmission probability? Can an increase in learning time caused by synaptic depression always be compensated by increasing the population of active cells, or by elevating their spiking rates? The topological model permits addressing these questions computationally, at a phenomenological level, thus allowing us to move beyond mere correlative descriptions to a deeper understanding of the spatial memory deterioration mechanisms.

The hypothesis about topological nature of the hippocampal map^8^ is broader than the proposed Algebraic Topology (AT) model or the scope of questions that this model allows addressing. For example, the description based on the AT algorithms does not capture biologically relevant metrical differences between topologically equivalent paths or qualitative differences between topologically equivalent environments, e.g., between the widely used W-, U- or T-mazes, even though such differences are reflected in the place cell spiking patterns and are known to affect animals behavior^78–80^. Addressing these differences requires using alternative mathematical apparatuses, e.g., Qualitative Space Representation (QSR) techniques, such as Region Connection Calculi (RCC)^81–83^, which would complement the scope of topological methods used in neuroscience^30^. In the current approach, we use AT instruments to assess a particular scope of questions, namely to estimate the conditions that guarantee structural integrity of the cognitive map and to describe its overall topological shape.

Fundamentally, producing a cognitive map requires two key components: a proper temporal structure of the spike trains and a physiological mechanism for detecting and interpreting neuronal coactivity–a suitable network architecture, a proper distribution of the connectivity strengths, of the parameters of synaptic plasticity, etc. All these components influence spike transmission and detection probabilities, which, in our model, affect the shape and topological structure of the coactivity complex, the statistics of learning times, the structure of the learning region, etc. This produces a quantitative connection between the information processed at the microscopic level (neurons and synapses) and the properties of the large-scale representations of space emerging at the organismal level, described here by means of the Persistence Homology theory^84–86^.

**Figure 9:**
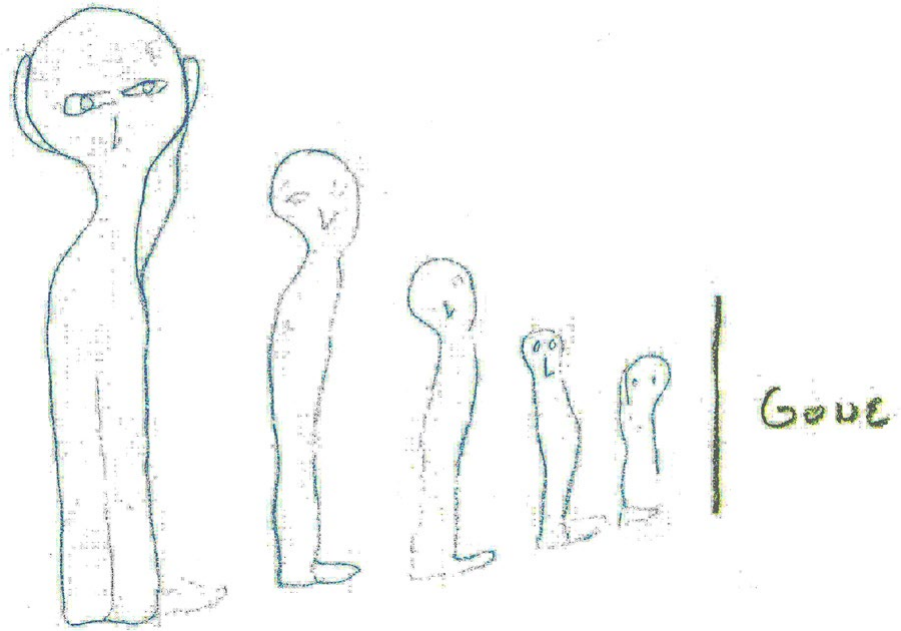
A drawing by an Alzheimer disease patient depicting his own perception of the disease’s development. The word to the right of the vertical bar is “Gone”.

Lastly, it should be pointed out that the low-dimensional components of the coactivity complexes were used above to represent cognitive maps, i.e., frameworks spatial memories. However, the combinations of the coactive place cells, modeled as simplexes of ${{\mathscr{T}}}_{{\rm{eff}}}$, may represent generic memory elements^87,88^. In other words, it can be argued that the net structure of ${{\mathscr{T}}}_{{\rm{eff}}}$ represents not only spatial, but also nonspatial memories–a larger memory framework that can be viewed as a “memory space”^89,90^. Thus, a disintegration of the cell assembly complex caused by deteriorating synapses discussed above may also be viewed as a model of the full memory space decay. From such perspective, it may be noticed that the results of the model parallel the experience of patients acquiring a slowly progressing dementia. For example, the model provides an explanation for the reason cognitive declines often do not manifest until quite a lot of damage has occurred. It also predicts that when the weakening synapses deteriorate beyond the range of parameters within which learning is effective, the damages push the neuronal ensemble beyond the bounds of the learning region. As a result, the failure becomes more frequent, and finally, the brain cannot perform that particular learning task, certain memories or abilities begin to flicker and then are lost mostly for good (Fig. 9).

## Methods

The computational algorithms used in this study were described in^12,13^:

The simulated environment shown on Fig. 1A is designed similarly to the arenas used in typical electrophysiological experiments. Combining such small arenas allows simulating learning in larger, more complex environments^13^. The simulated trajectory represents non-preferential, exploratory spatial behavior, with no artificial patterns of moves or favoring of one segment of the environment over another.

Place cell spiking probability was modeled as a Poisson process with the rate

$${\lambda }_{c}(r)={f}_{c}{e}^{-\frac{{(r-{r}_{c})}^{2}}{2{s}_{c}^{2}}}$$

where *f*_*c*_ is the maximal rate of place cell *c* and *s*_*c*_ defines the size of its place field centered at *r*_*c*_ = (*x*_*c*_, *y*_*c*_)^91^. In an ensemble of *N* place cells, the parameters *s*_*c*_ and *f*_*c*_, are log-normally distributed with the means *f* and *s* and the variances *σ*_*f*_ and *σ*_*s*_. To avoid overly broad or overly narrow distributions, we used additional conditions *σ*_*f*_ = *af* and *σ*_*s*_ = *bs*, with *a* = 1.2 and *b* = 1.7^12^. In addition, spiking probability was modulated by the *θ*-wave^13,92,93^. The *θ*-wave also defines the temporal window *w* ≈ 250 ms (about two *θ*-periods) for detecting the place cell spiking coactivity, as suggested by experimental studies^92–94^ and by our model^13^. This value also defines the timestep used in the computations. The place field centers *r*_*c*_ for each computed place field map were randomly and uniformly scattered over the environment.

Place cell ensembles are specified by a triple of parameters (*s*, *f*, *N*) and hence the learning region represents a domain of this 3*D* parameter space. The ensembles studied above contain between *N* = 50 and *N* = 400 place cells. The ensemble mean peak firing rate *f* ranges from 4 to 40 Hz, and the average place field size ranges between ~12 cm and ~90 cm ($4\le s\le 30$ cm).

Persistent Homology Theory is used to describe the evolving topological shape of the coactivity complexes in terms of their homological invariants^84–86^. In particular, it allows computing the time-dependence of the Betti numbers and deducing the dynamics of its topological loops–their mean lifetimes, their mean lengths, their numbers, etc. Computations were performed using javaplex computational software developed at Stanford University^95^.
